# Abundance of bacteria and histopathologic findings in the small intestinal mucosa of dogs with chronic inflammatory enteropathies

**DOI:** 10.1371/journal.pone.0337930

**Published:** 2025-12-22

**Authors:** Cintia R. R. Queiroz-Machado, Mohsen Hanifeh, Enrico Bottero, Chi-Hsuan Sung, Patricia Eri Ishii, Roberto M. C. Guedes, Raphael R. Wenceslau, Susanne Kilpinen, Thomas Spillmann, Jonathan A. Lidbury, Joerg M. Steiner, Jan S. Suchodolski, Paula R. Giaretta

**Affiliations:** 1 Veterinary School, Universidade Federal de Minas Gerais, Belo Horizonte, Minas Gerais, Brazil; 2 University of Helsinki, Helsinki, Finland; 3 Endovet Group, Roma, Italy; 4 Gastrointestinal Laboratory, Department of Small Animal Clinical Sciences, College of Veterinary Medicine and Biomedical Sciences, Texas A&M University, College Station, Texas, United States of America; Chattogram Veterinary and Animal Sciences University, BANGLADESH

## Abstract

Chronic inflammatory enteropathy (CIE) is often retrospectively classified as food-responsive, steroid-responsive, and antibiotic-responsive enteropathy. However, whether bacterial fluorescence *in situ* hybridization or histopathologic findings can predict treatment response has not been extensively investigated. The study aimed to investigate the relationship between clinical disease activity, bacterial abundance, and histopathologic scores in the small intestine of dogs with different subtypes of CIE. Samples from the duodenum and ileum from 54 dogs with different categories of CIE and 11 control dogs were used for investigation of bacterial abundance with fluorescence *in situ* hybridization and histopathologic changes. Duodenal bacterial abundance did not differ among the four groups. While the abundance of total superficial bacteria and attached bacteria was increased in the ileal mucosa of dogs with antibiotic-responsive enteropathy compared to control dogs, it was not significantly different between the CIE groups. Summative histopathologic scores did not differ between the different CIE categories. The histopathologic findings, including neutrophilic inflammation, were variable and most of the parameters overlapped between the different CIE. There was a positive correlation between duodenal and ileal histopathologic scores and the canine inflammatory bowel disease activity index. In summary, increased bacterial abundance and histopathologic scores were found in CIE compared to healthy dogs, but these findings could not predict the treatment response for the different categories of CIE. Fluorescence *in situ* hybridization for bacteria in small intestinal biopsies had limited utility in distinguishing between different CIE types.

## Introduction

Chronic inflammatory enteropathy (CIE) in dogs is a condition of unknown origin, characterized by persistent gastrointestinal (GI) signs lasting over three weeks, exclusion of identifiable causes, and histologic evidence of enteritis [1–3]. It is classified based on treatment response: food-responsive (FRE), steroid/immunosuppressant-responsive (SRE), antibiotic-responsive (ARE), and non-responsive [4]. While pathogenesis remains unclear, bacterial overgrowth or a single causative microorganism has not been proven [5,6], though antimicrobials are often used after failure of an elimination diet [7,8]. However, the efficacy and role of antimicrobials in CIE have been questioned due to concerns about bacterial resistance [8,9] and gut microbiome disruption [10–12]. Antimicrobial effects in ARE cases are often short-lived, with frequent relapses [1,9,13,14]. This raises concerns about prolonged dysbiosis and its impact on treatment outcomes [12,15]. In line with these concerns, the use of antibiotics is declining and being replaced by microbiota modulation strategies [16]. However, despite recent advances, many general practitioners still rely on antibiotic therapy for the management of CIE in clinical settings. Accordingly, new terminology has been proposed to replace the term “antibiotic-responsive” enteropathy, such as “idiopathic intestinal dysbiosis” [17] and “microbiota-modulation-responsive enteropathy” [16].

Investigating mucosal bacterial populations in situ helps to better understand their role in CIE [18]. Fluorescence in situ hybridization (FISH) enables spatial analysis of bacterial load [19], though previous studies show no difference in total bacterial abundance between CIE and control dogs in colonic and ileal mucosa [5,19,20]. However, FISH has revealed differences in bacterial composition in specific intestinal compartments [5,19,20]. While some diagnostic services offer FISH to detect invasive bacteria, its clinical relevance beyond *Escherichia coli*-associated granulomatous colitis is uncertain. Before the standardization of histopathologic criteria by the World Small Animal Veterinary Association Gastrointestinal Standardization (WSAVA) [21], a few studies have compared the intestinal lesions between dogs with FRE and SRE and detected no significant differences in histopathologic scores [22–24], but no studies have yet characterized mucosal microbiota across the three CIE categories or used FISH on duodenal samples. Besides that, the detailed histopathologic phenotype of the three clinical outcomes needs to be better characterized, and studies comparing them utilizing the WSAVA histopathologic standards are nonexistent.

This study aimed to investigate bacteria in the small intestinal mucosa of healthy dogs and those with different types of CIE, using samples obtained before the final diagnosis and treatment. We analyzed the relationship between bacterial abundance, histopathologic scores, and clinical disease activity. Clinicians often prescribe antimicrobials for dogs with neutrophilic inflammation in intestinal biopsies, suspecting an underlying bacterial infection. We explored whether dogs with ARE would exhibit higher neutrophilic inflammation and bacterial loads in the small intestinal mucosa compared to FRE or SRE dogs. Our findings provide insight into the limited utility of histopathological findings and FISH results in predicting the treatment response for the different categories of CIE.

## Materials and methods

### Ethics statement

This study was carried out following the Finnish National Animal Experiment Board (protocols: ESLH-2007–09833/Ym-23, ESAVI2010–04178/Ym-23, and SAVI/7290/04.10.03/2012), and the Texas A&M Institutional Animal Care and Use Committee (protocols 2012−083 and 2015−0069). The Institutional Animal Care and Use permit used for the control group was approved by the Ethics Committee on Animal Use of the United Metropolitan Colleges University Center (protocol 4079150419). Written informed consent was obtained from all owners of dogs.

### Animal population and samples

A retrospective study with convenience samples was performed. Archived intestinal mucosal biopsy samples of dogs in which CIE that had been diagnosed at the University of Helsinki (Finland), Endovet Group (Italy), and through an open enrollment of dogs with CIE through the Gastrointestinal Laboratory, Texas A&M University (USA) were used. Formalin-fixed paraffin-embedded small intestinal tissue samples from 54 dogs with CIE and 11 control dogs were used. None of the dogs received antimicrobials or corticosteroids in the month before sample collection. For the CIE group, inclusion criteria were GI signs such as vomiting, diarrhea, tenesmus, hematochezia, and/or weight loss lasting longer than 3 weeks with histologic evidence of intestinal inflammation and exclusion of specific causes of GI signs. Diagnostic tests were performed at the discretion of the attending veterinarian and typically consisted of a complete blood count and serum chemistry (alanine transaminase and alkaline phosphatase activities, urea, creatinine, glucose, total protein, albumin, sodium, potassium, cobalamin, folate, trypsin-like immunoreactivity). Other examinations included urinalysis (specific gravity, dipstick, and sediment), fecal examination for parasites, bacterial culture, abdominal ultrasound, and gastroduodenoscopy/colonoscopy. Basal serum cortisol concentration was evaluated in 24 dogs with CIE to rule out hypoadrenocorticism. The treatment response was determined by the attending veterinarian after intestinal biopsies were obtained and consisted of a reduction of at least 50% of the canine inflammatory bowel disease activity index (CIBDAI) [25] after treatment or an improvement of the fecal consistence scores from >3 to ≤3 [26,27]. Dogs in the FRE group responded to a 2–4 week dietary trial with commercial hydrolyzed protein diet or novel protein diet alone. Dogs that had an unsuccessful dietary trial and required treatment with an immunomodulatory drug (e.g., glucocorticoids or other immunomodulatory drug) for improvement of the clinical signs were classified as SRE. Dogs with a clinical diagnosis of protein-losing enteropathy that did not respond to immunomodulatory drugs were not included in this study. Dogs with ARE showed improvement of the clinical signs within three days after administration of an antimicrobial drug (e.g., tylosin or metronidazole) and had a relapse of the clinical signs when antimicrobials were discontinued [26]. Long term follow-up data was available for 21/54 dogs, with a median of 9 months of follow-up time (range: 2–27 months). All cases with follow-up information remained responsive to the same initial treatment. Duodenal and ileal formalin-fixed, paraffin embedded (FFPE) tissue samples were retrieved from the archive and included a minimum of four good quality endoscopic biopsies, ranging from 4 to 21 biopsies. These samples were obtained through endoscopy before the treatment as part of the routine diagnostic procedures at the discretion of the attending veterinarian, fixed in 10% formalin, routinely processed for histology, stained with hematoxylin and eosin (HE), and scanned at 400x magnification.

Archived FFPE endoscopic samples from duodenum and ileum from healthy dogs from a previous unpublished study were used as a control group. The control group consisted of 11 client-owned adult dogs that were considered healthy based on absence of GI signs for the past 6 months, a negative fecal flotation for parasites, and unremarkable histopathologic findings, blood count, serum chemistry profile, serum cobalamin, folate, trypsin-like immunoreactivity, pancreatic lipase immunoreactivity, basal cortisol, fecal enteropathogen panel, and fecal dysbiosis index.

### Histopathologic examination and clinical disease activity

Histopathologic examination and scoring of lesions were performed by a single board-certified veterinary pathologist (PRG) experienced in GI pathology. The pathologist was blinded to CIE and control groups. The digitally HE scanned slides were systematically evaluated, and lesions were graded following the histopathologic scoring system developed by the WSAVA Standardization Group [21]. Morphologic parameters (i.e., villous stunting, epithelial injury, crypt dilation/distortion, lacteal dilatation, mucosal fibrosis) and inflammatory parameters (i.e., intraepithelial lymphocytes, lamina propria eosinophils, lamina propria lymphocytes and plasma cells, lamina propria neutrophils, lamina propria macrophages) were evaluated and scored as absent (0), mild (1), moderate (2), and marked (3) for the duodenum and the ileum separately. For each parameter, the highest score observed across the samples was recorded. Finally, the summative histopathologic score for each intestinal segment was calculated by summing the scores for the ten parameters. Duodenal samples were available in 11/11 control dogs, 13/13 FRE dogs, 12/18 SRE dogs, and 15/23 dogs with ARE. Samples from the ileum were available in 10/11 control dogs, 5/13 FRE dogs, 10/18 SRE dogs, and 11/23 ARE dogs.

The CIBDAI is a numeric scoring system based on six clinical signs: attitude/activity, appetite, vomiting, fecal consistency, frequency of defecation, and weight loss. The total score is classified as clinically insignificant (0–3), mild (4–5), moderate (6–8), or severe (9 or greater) clinical disease activity [25]. The severity of clinical disease activity at diagnosis was assessed at the time of endoscopy for 32/54 dogs and retrospectively calculated for 22/54 dogs using the CIBDAI.

### FISH and image analyses

The small intestinal samples from duodenum and/or ileum were routinely processed for histology, and 5 µm formalin-fixed paraffin-embedded sections were used for FISH, as previously described [5,19] with modifications (see S1 Text). The EUB338 probe [28] targeting the 16S rRNA gene of bacteria was used for total bacterial counts and 5’-labeled with Cy3 [28]. Sections of intestine with salmonellosis were used as the positive control, while normal lung was used as the negative control. In selected control dogs and dogs with CIE, sections were hybridized with the irrelevant probe non-EUB338, as a negative control. Additionally, slides from a case of neonatal piglet diarrhea associated with enteroadherent *Enterococcus hirae*, *Clostridium piliforme* infection in a cat, as well as sections from a case of swine enteric colibacillosis, were used to validate the sensitivity of the probe and protocol.

**Fig 1:**
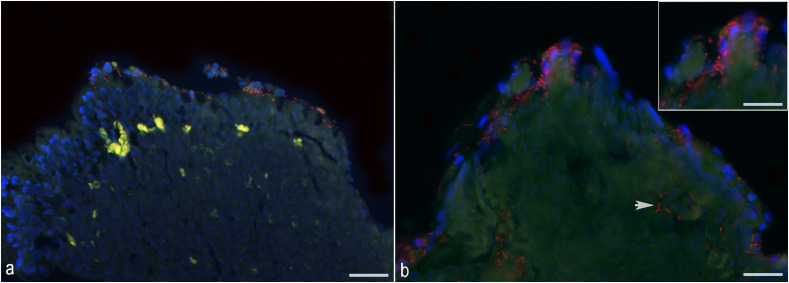
Fluorescence *in situ* hybridization with EUB338 probe in ileal samples from dogs with chronic inflammatory enteropathy before treatment. Red color represents labeled bacteria; the autofluorescence of the intestinal mucosa and contents is represented in green; autofluorescence of erythrocytes in blood vessels and presumed lipofuscin granules in the lamina propria is represented in yellow to neon green; DAPI-stained nuclei of ileal mucosa are represented in blue. **(a)** Bacteria are attached to the superficial epithelium or associated with superficial cellular debris of the ileum in a dog with antibiotic-responsive enteropathy. **(b)** Aggregates of labeled bacteria invasive (arrow) are in the lamina propria of the ileum of a dog with food-responsive enteropathy. The disorganization, unalignment and loss of DAPI-stained nuclei of the superficial epithelium indicates epithelial erosion with numerous attached bacteria, highlighted in the inset. Scale bar = 20 µm. Inset scale bar = 15 µm.

The tissue sections were analyzed using a Leica DM4000 B fluorescence microscope. For each case and intestinal segment (duodenum and ileum), all available biopsy fragments were evaluated. Ten random 400x fields with labeled bacteria on the mucosal surface were photographed for analysis, including a minimum of four endoscopic biopsies. Each field was captured with the DAPI filter for the identification of host cell nuclei, fluorescein isothiocyanate (FITC) filter for background autofluorescence from the host epithelium, and tetramethylrhodamine-isothiocyanate (TRITC) filter for identification of probe-labeled bacteria. Bacterial quantification was performed using ImageJ 1.46 r software. All pixels representing labeled bacteria for each image were recorded as total bacteria, and the average area in pixels corresponding to bacteria was calculated for each slide. Additionally, the spatial distribution of attached bacteria (i.e., those juxtaposed to the epithelium) and invasive bacteria (i.e., those located in the lamina propria) was recorded. Average area in pixels corresponding to invasive bacteria within the mucosa or attached to the epithelium for each image were calculated when bacteria were present in these compartments. Only labeled bacteria closely juxtaposed to the surface epithelium as demonstrated in the inset of Fig 1B were considered as attached bacteria for the purpose of quantification, while only strong positive hybridization signals within the lamina propria were counted as invasive bacteria within the mucosa (Fig 1B). Additional information on image analysis can be found in the S1 Text.

### Statistical analyses

The average area in pixels of labeled bacteria per slide, bacteria attached to the epithelium, invasive bacteria within the mucosa, summative histopathologic scores, individual histopathologic scores, CIBDAI, and serum albumin concentrations were compared among four groups (healthy controls, FRE, SRE, and ARE, respectively) using Kruskal-Wallis tests, followed by Dunn’s multiple comparisons test. Data distribution was assessed using the Shapiro–Wilk test, which indicated that the data were not normally distributed. The relationships between summative histopathologic score, the average area in pixels of labeled bacteria, and the CIBDAI score were evaluated by Spearman’s correlation tests using the Spearman’s correlation coefficient (ρ). The average area in pixels of total labeled bacteria per slide, bacteria attached to the epithelium, and invasive bacteria within the mucosa were compared between dogs with hypoalbuminemia and without hypoalbuminemia using Mann-Whitney U tests. Data analyses were performed using the PMCMRplus package (version 1.9.12) in the R 4.1.3 statistical software [29] and GraphPad Prism version 10.0.0 for Windows, GraphPad Software. We further investigated differences in the frequency (presence or absence) of neutrophils and eosinophils in the small intestinal lamina propria among groups based on a Fisher’s exact test. For all hypothesis tests, the significance level was set at 5%.

## Results

### Patient characteristics

Fifty-four client-owned dogs with CIE were further classified as FRE (n = 13), SRE (n = 18), or ARE (n = 23) according to treatment response. A brief description of the patients’ characteristics is presented in Table 1. Most of the dogs from the ARE group were treated with tylosin (19/23), while 3/23 received metronidazole, and one dog received both tylosin and metronidazole. Serum albumin concentrations were lower in dogs with SRE (median, 2.7 g/dL; range, 1.1–3.5 g/dL) compared to dogs with FRE (median, 3.2 g/dL; range, 2.8–3.7 g/dL; *P* = 0.02).

**Table 1:** Summary of the sample population’s characteristics.

Characteristic	Control (n = 11)	FRE (n = 13)	SRE (n = 18)	ARE (n = 23)
N. males/females	7/4	5/8	11/7	19/4
Breed most represented (n)	Mixed-breed (4)	German Shepherd (3)	German Shepherd (3)	German Shepherd (5)
Median age (range) in years	3 (1-9)	4 (1-9)	3 (1-11)	7 (1-12)
Median body weight (range) in kg	8 (2.6-14.8)	21.1 (7.0-49.2)	14.6 (4.3-63)	24.8 (3-42)
Median CIBDAI score (range)	0	4 (2-9)	6 (1-16)	5 (1-11)
N. of insignificant disease activity cases	0	3	5	9
N. of mild disease activity cases	0	6	2	4
N. of moderate disease activity cases	0	3	6	6
N. of severe disease activity cases	0	1	5	4
Median summative histopathologic score (range)	0 (0-2)	4 (1-10)	4 (1-16)	2 (0-12)
Median (range) serum albumin values in g/dL	NA	3.2 (2.8-3.7)	2.7 (1.1-3.5)	3.2 (1.9-3.5)
N. of hypoalbuminemic^*^ cases (median; range) of serum albumin values in g/dL of hypoalbuminemic cases	NA	3 (2.8; 2.8-2.8)	12 (2.5; 1.1-2.9)	9 (2.5; 1.9-2.9)

FRE, food-responsive enteropathy; SRE, steroid-responsive enteropathy; ARE, antibiotic-responsive enteropathy; N., number; NA not available; *, serum albumin concentration < 3.0 g/dL. Serum albumin values were available for 22/23 dogs with ARE.

### Bacterial quantification

**Fig 2:**
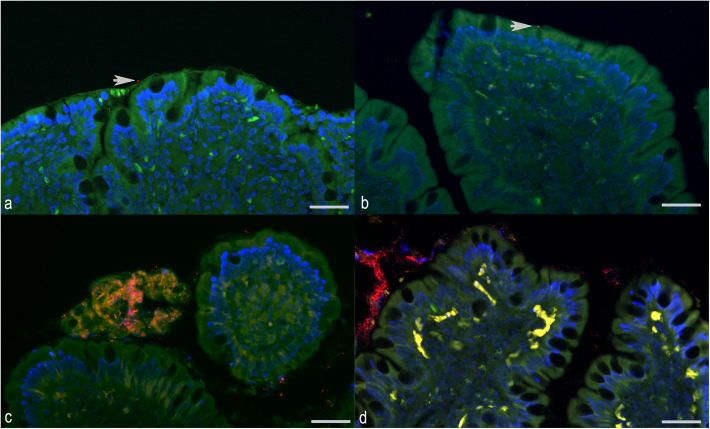
Fluorescence *in situ* hybridization with EUB338 probe in small intestinal samples from dogs with chronic inflammatory enteropathy before treatment. Red color represents labeled bacteria; the autofluorescence of the intestinal mucosa and contents is represented in green; autofluorescence of erythrocytes in blood vessels and presumed lipofuscin granules in the lamina propria is represented in yellow to neon green; DAPI-stained nuclei of ileal mucosa are represented in blue. **(a)** A single labeled bacterium (arrow) is on the mucosal surface (not attached) of the duodenum of a dog with steroid-responsive enteropathy. **(b)** A single labeled bacterium (arrow) is attached to the superficial epithelium of the duodenum of a dog with antibiotic-responsive enteropathy. **(c)** Numerous labeled bacteria are mainly associated with the superficial mucus in the ileum of a dog with antibiotic-responsive enteropathy. **(d)** Abundant bacteria are mainly associated with the superficial mucus and debris cells in the ileum of a dog with food-responsive enteropathy. Scale bar = 20 µm.

**Fig 3:**
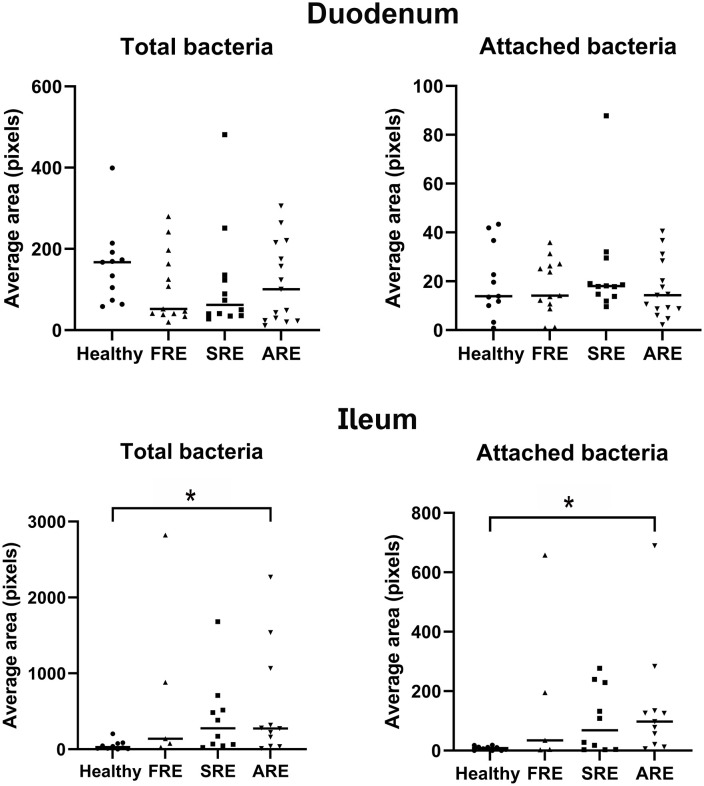
Comparison of the average area in pixels corresponding to total bacteria and the average area in pixels of bacteria attached to the surface epithelium between healthy dogs and dogs with different forms of chronic inflammatory enteropathy in both the duodenum and the ileum. Although dogs with ARE have increased loads of total and attached bacteria in the ileum than healthy dogs, there is no difference between the three diseased groups. Dunn’s multiple comparisons Test. FRE, food-responsive enteropathy; SRE, steroid-responsive enteropathy; ARE, antibiotic-responsive enteropathy. *P < 0.05.

The average area in pixels corresponding to total EUB338-labeled bacteria detected in the duodenum (Fig 2 and 3) was not significantly different among the four groups. The average area in pixels corresponding to attached bacteria in the duodenum was not significantly different among the four groups. In the ileum (Fig 2 and 3), dogs with ARE had a higher average (*P *= 0.04) area in pixels of total bacteria than the healthy control dogs; however, there were no statistical differences between dogs with ARE and FRE or SRE. The average area in pixels of attached bacteria in the ileum was higher in dogs with ARE (*P* = 0.009) compared to healthy control dogs; however, it did not differ from those in other CIE groups. Most of the samples did not have bacteria within the mucosa, and average area in pixels of invasive bacteria within duodenal and ileal lamina propria showed no significant differences among the four groups. Mann-Whitney U tests revealed no significant differences in bacterial abundance in the duodenum or ileum between dogs with or without hypoalbuminemia, or between German Shepherds and non-German Shepherd dogs with CIE. A summary of the results of quantification of the area corresponding to bacterial area in pixels is presented in the S1 Table.

### Histopathologic scores and clinical disease activity

**Fig 4:**
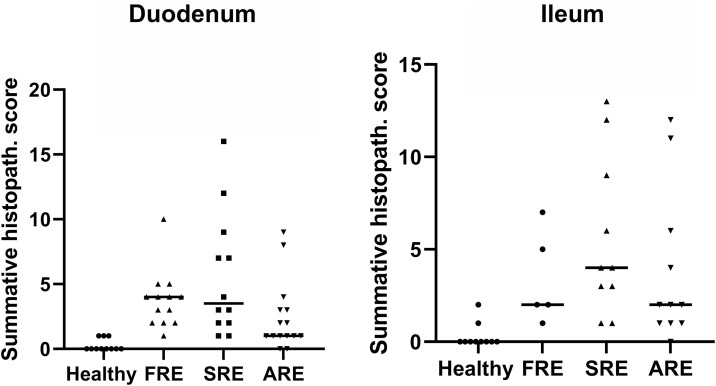
Summative histopathologic scores of biopsies from the ileum and duodenum collected before treatment. There is no distinction between the three groups of dogs with chronic inflammatory enteropathy. Dunn’s multiple comparisons Test.

The ileal summative histopathologic scores were higher in dogs with SRE (adjusted *P* < 0.001) and ARE (adjusted *P *= 0.02) when compared to healthy control dogs (Fig 4). Dogs with FRE, SRE, or ARE had higher duodenal summative histopathologic scores (adjusted *P* < 0.001, < 0.001, and = 0.04, respectively) than healthy control dogs. Moreover, there was no statistically significant difference among the three CIE groups for summative histopathologic scores in both small intestinal anatomical sites. There was no statistical difference in CIBDAI scores among CIE groups.

Evaluation of selected histopathologic parameters showed significant differences among groups in both the duodenum (Table 2) and the ileum (Table 3). The parameters encompassed morphologic changes (mucosal fibrosis and epithelial injury) as well as changes in the type of mucosal cellular infiltrate in CIE groups. Dogs with SRE had significantly higher scores for surface epithelial injury in the ileum than dogs with ARE (*P* = 0.02) or healthy control dogs (*P* = 0.001). Epithelial erosion was observed in the duodenum and/or ileum in 10/54 dogs with CIE. This included 6 dogs with SRE, 2 with FRE, and 2 with ARE. Dogs with SRE (*P *< 0.001) or FRE (*P *= 0.04) had higher ileal lamina propria neutrophil scores when compared to healthy control dogs. The ileal neutrophil scores were higher in dogs with SRE when compared to dogs with ARE (*P *= 0.02).

**Table 2:** Duodenal histopathologic scores of the studied groups of chronic inflammatory enteropathy based on the WSAVA guidelines.

Parameter	Control (N = 11)			FRE (N = 13)			SRE (N = 12)			ARE (N = 15)			*P-*value*
	Median	IQR	Min-Max	Median	IQR	Min-Max	Median	IQR	Min-Max	Median	IQR	Min-Max	
Epithelial injury	0	0−0	0-1	0	0-1	0-2	0	0-2	0-2	0	0−0	0-1	0.15
Villous stunting	0	0−0	0−0	0	0−0	0−0	0	0-0.5	0-2	0	0−0	0-2	0.07
Crypt dilation	0	0−0	0−0	0	0−0	0-1	0	0−0	0-2	0	0−0	0-1	0.51
Lacteal dilation	0	0−0	0-1	0	0−0	0-1	0	0-1.25	0-3	0	0−0	0-1	0.44
Mucosal fibrosis	**0** ^**a**^	0−0	0−0	**0** ^**a**^	0−0	0−0	**0** ^**b**^	0-0.25	0-2	**0** ^**a**^	0−0	0−0	**0.01**
IEL	0	0−0	0−0	0	0−0	0-1	0	0-1	0-1	0	0−0	0-2	0.13
LP	**0** ^**a**^	0−0	0-1	**1** ^**b**^	0-2	0-2	**1** ^**bc**^	0-1	0-3	**0** ^**ac**^	0-1	0-2	**0.005**
Eosinophils	**0** ^**a**^	0−0	0−0	**1** ^**b**^	0-1	0-2	**0** ^**b**^	0-1	0-2	**0** ^**ab**^	0-0.5	0-1	**0.02**
Neutrophils	**0** ^**a**^	0−0	0−0	**1** ^**b**^	0-1	0-2	**1** ^**b**^	0.75-1	0-2	**1** ^**b**^	0-1	0-1	**0.001**
Macrophages	0	0−0	0−0	0	0−0	0−0	0	0−0	0−0	0	0−0	0-1	0.50
Sum. Histopath. Score	**0** ^**a**^	0-0.5	0-1	**4** ^**b**^	2-4	1-10	**3.5** ^**b**^	2-7.5	1-16	**1** ^**b**^	1-3	0-9	**< 0.001**

WSAVA, World Small Animal Veterinary Association Gastrointestinal Standardization; FRE, food-responsive enteropathy; SRE, steroid-responsive enteropathy; ARE, antibiotic-responsive enteropathy; IQR, interquartile range; IEL, intraepithelial lymphocytes; LP, lymphocytes and plasma cells; Sum. Histopath. Score, summative histopathologic scores; * *P* values resulted from Kruskal-Wallis Test; Median values superscripted with different letters indicate significant differences (*P* < 0.05) between the groups of the same row (Pairwise comparisons; Dunn’s Test).

**Table 3:** Ileal histopathologic scores of the studied groups of chronic inflammatory enteropathy based on the WSAVA guidelines.

Parameter	Control (N = 10)			FRE (N = 5)			SRE (N = 10)			ARE (N = 11)			*P*-value*
	Median	IQR	Min-Max	Median	IQR	Min-Max	Median	IQR	Min-Max	Median	IQR	Min-Max	
Epithelial injury	**0** ^**a**^	0−0	0−0	**0** ^**ab**^	0-1	0-2	**1** ^**b**^	0.25-1.75	0-2	**0** ^**a**^	0−0	0-2	**0.007**
Villous stunting	0	0−0	0−0	0	0-1	0-1	0	0-1	0-2	0	0−0	0-1	0.12
Crypt dilation	0	0−0	0−0	0	0−0	0−0	0	0−0	0−0	0	0−0	0-1	0.53
Lacteal dilation	0	0−0	0-2	0	0-1	0-1	1	0-1	0-2	1	0-1	0-2	0.39
Mucosal fibrosis	0	0−0	0−0	0	0−0	0−0	0	0−0	0−0	0	0−0	0-1	0.53
IEL	0	0−0	0−0	0	0−0	0-1	0.5	0-1	0-3	0	0−0	0-3	0.07
LP	0	0−0	0−0	0	0−0	0-2	0	0-0.75	0-2	0	0-1	0-2	0.24
Eosinophils	0	0−0	0−0	0	0-1	0-1	0	0-1	0-2	0	0-1	0-2	0.16
Neutrophils	**0** ^**a**^	0−0	0−0	**1** ^**bc**^	0-1	0-1	**1** ^**b**^	1−1	0-2	**0** ^**ac**^	0-1	0-2	**< 0.001**
Macrophages	0	0−0	0−0	0	0−0	0−0	0	0-0.75	0-2	0	0−0	0-1	0.18
Sum. Histopath. Score	**0** ^**a**^	0−0	0-2	**2** ^**ab**^	2-5	1-7	**4** ^**b**^	3-8.25	1-13	**2** ^**b**^	1-5	0-12	**< 0.001**

FRE, food-responsive enteropathy; SRE, steroid-responsive enteropathy; ARE, antibiotic-responsive enteropathy; IQR, interquartile range; IEL, intraepithelial lymphocytes; LP, lymphocytes and plasma cells; Sum. Histopath. Score, summative histopathologic scores; * *P* values resulted from Kruskal-Wallis Test; Median values superscripted with different letters indicate significant differences (*P* < 0.05) between the groups of the same row (Pairwise comparisons; Dunn’s Test).

In the duodenum, all the CIE groups, FRE (*P* = 0.01), SRE (*P* < 0.001), and ARE (*P *= 0.01), had higher neutrophilic infiltrate scores than healthy control dogs. SRE and FRE groups had higher scores for lamina propria eosinophils (*P *= 0.03 and = 0.004, respectively) and lamina propria lymphocytes and plasma cells (*P *= 0.01 and = 0.002, respectively) compared to healthy control dogs. In addition, lamina propria lymphocytes and plasma cells scores were higher in dogs with FRE when compared to dogs with ARE (*P* = 0.04). Mucosal fibrosis scores in the duodenum were significantly higher in dogs with SRE than those in FRE (*P* = 0.008), ARE (*P* = 0.006), or healthy control (*P *= 0.01) dogs. Other histopathologic parameters showed no significant differences among those groups (Table 2).

A Fisher’s exact test showed that neutrophils were found more often in the lamina propria of the ileum (*P* < 0.001) of dogs with FRE (3/5; 60%) or SRE (9/10; 90%) compared to healthy control dogs (0/10; 0%). Neutrophils were more likely to be present in the ileum of dogs with SRE than those with ARE (4/11; 36%). Also, an eosinophilic infiltrate was more likely to be present in the duodenum (*P *= 0.02) of dogs with FRE (7/13; 54%) and SRE (5/12; 42%) than in healthy control dogs (0/10; 0%). However, the frequency of eosinophilic inflammation in the ileum did not differ between the four groups (*P* = 0.10) (Table 4).

**Table 4:** Frequency of neutrophils and eosinophils in the ileum and duodenum of the studied groups of chronic inflammatory enteropathy.

	Ileum					Duodenum				
	Control	FRE	SRE	ARE	*p*value	Control	FRE	SRE	ARE	*P*-value
Neutrophils	0%^**a**^ (0/10)	60%^**bc**^ (3/5)	90%^**b**^ (9/10)	36%^**ac**^ (4/11)	<0.001	0%^**a**^ (0/11)	54%^**b**^ (7/13)	75%^**b**^ (9/12)	53%^**b**^ (8/15)	0.001
Eosinophils	0%^a^ (0/10)	40%^a^ (2/5)	40%^a^ (4/10)	36%^a^ (4/11)	0.10	0%^a^ (0/11)	54%^b^ (7/13)	42%^b^ (5/12)	27%^ab^ (4/15)	0.02

FRE, food-responsive enteropathy; SRE, steroid-responsive enteropathy; ARE, antibiotic-responsive enteropathy. *P* values resulted from Fisher’s Exact Test. The frequency values superscripted with different letters indicate significant differences (*P* < 0.05) between the groups of the same row of each organ (Pairwise comparisons of proportions; Fisher Test).

### Correlations

**Fig 5:**
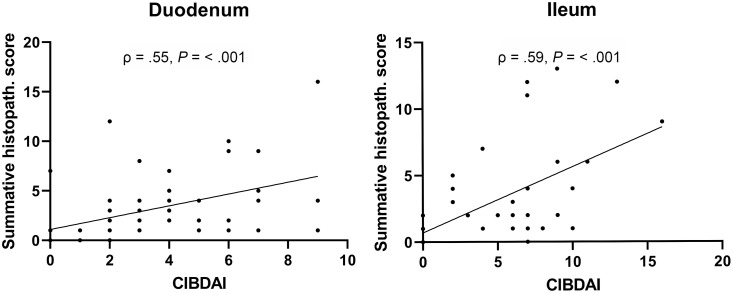
Positive correlation between the canine inflammatory bowel disease activity index (CIBDAI) and summative histopathologic scores in samples from duodenum and ileum collected before treatment. ρ, Spearman’s correlation coefficient.

Correlations between summative histopathologic scores, bacterial area in pixels, and CIBDAI scores were investigated. No significant correlation was detected between the summative histopathologic score and mucosal bacteria in the duodenum and ileum. However, there was a significant positive correlation between the CIBDAI score and the ileal (ρ = 0.59, *P *< 0.001) or duodenal (ρ = 0.55, *P* < 0.001) histopathologic scores (Fig 5). There was a negative correlation between albumin concentration and lacteal dilation in both the duodenum (ρ = −0.34, *P* = 0.02) and ileum (ρ = −0.66, *P* = 0.003) of dogs with CIE.

## Discussion

Our results showed no significant differences in bacterial load in the duodenal mucosa among the different CIE categories, and no evidence of bacterial overgrowth in the duodenum as a feature of the condition of dogs with ARE. ARE was initially described as a chronic enteropathy affecting mainly German Shepherd dogs, thought to be caused by bacterial overgrowth in the proximal small intestine and historically called small intestine bacterial overgrowth [30]. However, a later study found that dogs with ARE had similar or lower bacterial counts in duodenal juice cultures compared to dogs with other enteropathies [6]. Our findings align with these later studies, show no increased bacterial load in the duodenal mucosa of dogs with ARE, FRE, or SRE compared to healthy controls.

The use of antimicrobials in dogs with CIE has raised concerns about antimicrobial resistance, intestinal dysbiosis, and frequent relapses after discontinuation [4,19,31]. Significant changes in gut and fecal microbiota have been reported in CIE dogs after antimicrobial therapy [8,9]. The higher bacterial load and number of attached bacteria found in the ileum of dogs with ARE compared to healthy controls may indicate more pronounced dysbiosis in this group. However, there was considerable overlap between CIE groups, with no differences in mucosal bacteria between dogs with ARE, FRE, and SRE. A previous sequencing-based study found no distinct characteristics in the fecal microbiota of dogs with ARE [13]. Although none of the dogs in our study had received antibiotics in the month before sample collection, prior antimicrobial treatments may occasionally result in long-term changes in the intestinal microbiota [12].

Most studies on the intestinal microbiota in dogs with CIE are sequencing-based and focus on fecal samples [18]. The mucosal microbiota has been previously evaluated in humans and animals with chronic enteropathies [5,19,32–35], and FISH has provided valuable insights in the study of microbial biogeography [5,19,36]. Studies comparing the abundance of bacteria in general on the colonic and/or ileal mucosa of healthy dogs and dogs with CIE found no evidence of an increased bacterial load [5,19,20]. One study has described decreased numbers of bacteria, especially *Helicobacter* spp. in the colonic crypts of dogs with CIE when compared to healthy dogs [19]. When evaluating specific bacteria, a study has demonstrated that dogs with SRE had increased *Enterobacteriaceae* bacteria attached onto surface epithelia of the colonic mucosa than healthy dogs [5]. Bacteria attached to the duodenal and ileal epithelial surface were observed in all groups of dogs in the present study, including healthy control dogs. *Lactobacillus* and *Bifidobacterium* genera, commensal bacteria of both humans and animals, can adhere to the intestinal tract and are the most common probiotic compositions [37]. The use of probiotics has not been reported in dogs of this study at the time of sampling. Attached bacteria are not necessarily pathogenic and may be part of the commensal microbiota. For example, enteroadherent *Enterococcus hirae* in kittens has been suggested to inhibit pathogenic *E. coli* attachment [38]. Therefore, identifying attached bacteria in histopathologic examination does not necessarily justify antimicrobial therapy. Avoiding unnecessary antimicrobials in dogs with CIE may help preserve the gut microbiota, allowing for alternative treatments like diet modification, prebiotics, and probiotics [17,31].

Bacterial invasion into the mucosa can trigger sustained intestinal inflammation [39], as seen in conditions like Crohn’s disease and ulcerative colitis in humans [40,41]. However, our study found no quantitative differences in invasive bacteria across CIE groups, with bacteria only detected in ulcerated areas of a few cases. Other research using FISH in dogs with CIE found significant bacterial invasion in the lamina propria only in cases of *E. coli*-associated granulomatous colitis [5]. Dogs with SRE (formerly referred to as inflammatory bowel disease) showed increased invasive bacteria in the colon, but not in the small intestine [5]. Additionally, a few healthy controls in our study had rare mucosal bacteria, suggesting that some intramucosal bacteria might result from artifacts introduced during biopsy sampling or processing.

Histopathologic evaluation of intestinal biopsies in dogs with CIE has traditionally been considered a limited diagnostic tool, primarily confirming inflammation and ruling out infections or neoplasia [22]. Typically, biopsies show non-specific inflammation and varying structural changes [14,22,42]. This study compared histopathologic findings across three CIE categories using the WSAVA histopathological score [21] and found no statistical differences in summative scores between groups for either small intestinal site. A prior study suggested that dogs with FRE may have normal duodenum histology, supporting the use of dietary trials in CIE cases with minimal histopathologic changes [43]. We observed that higher summative histopathologic scores in duodenum and ileum reflected greater clinical disease severity using CIBDAI, as previously described [44], although this correlation has not been consistently demonstrated in most studies.

Neutrophilic inflammation is commonly linked to infectious causes [45] and may lead clinicians to prescribe antimicrobials to eliminate pathogens [15]. Clinicians occasionally request FISH assays to investigate mucosal bacteria in cases with neutrophilic inflammation, though its clinical significance is unclear. Only dogs with FRE and SRE had a higher frequency of neutrophilic infiltrate in the ileum than controls, while dogs with ARE did not show higher neutrophilic inflammation despite increased bacteria in their ileal samples. Therefore, neutrophilic infiltrates alone do not justify antimicrobial use in dogs with CIE. Eosinophilic inflammation, often linked to food hypersensitivity [46,47], was significantly increased in dogs with FRE and SRE compared to healthy controls, suggesting that eosinophilic enteritis is not limited to food-responsive CIE. While a previous study found a tendency toward eosinophilic inflammation in FRE than other types of CIE [47], no significant differences in eosinophilic infiltrates between CIE groups were observed in our study. Eosinophilic enteritis is also common in steroid-responsive CIE [48] when parasites and FRE are ruled out [49], with other differential diagnoses including parasitic infections or intestinal pythiosis [50].

Lymphocytic and plasmacytic infiltration in the lamina propria is the most common finding in dogs with CIE [45,51]. These cells are known mediators of intestinal injury [52], with higher cytokine expression in dogs with CIE compared to healthy controls [53,54]. In our study, dogs with FRE and SRE had higher lymphocyte and plasma cell scores than healthy dogs, with no significant difference between the FRE and SRE groups. This aligns with previous studies showing similar numbers of CD3 lymphocytes in the duodenum [22,24,55] and frequency of lymphocytic-plasmacytic enteritis [54] in dogs with FRE and SRE. However, unlike earlier findings [55], dogs with FRE in our study had higher lymphocyte and plasma cell scores than those with ARE, possibly due to differences in diagnostic criteria, cell counting methods, and sampled populations between studies.

Mucosal architectural changes are considered to better reflect the clinical severity of GI disease compared to lamina propria infiltrates [17]. In our study, mucosal architectural changes, such as superficial epithelial injury and mucosal fibrosis, were more pronounced in dogs with SRE than other CIE groups. These findings are consistent with the more severe clinical manifestations often seen in SRE, including higher histopathologic scores and protein-losing enteropathy [1,56]. Consistent with the literature, our study showed that serum albumin concentrations in dogs with SRE were lower than in dogs with FRE. Crypt and lacteal dilation, villous stunting, epithelial injury [56] are associated with hypoalbuminemia in dogs with protein-losing enteropathy. Changes in enterocyte morphology have also been described in dogs with protein-losing enteropathy [57]. Several cases in our study that presented with hypoalbuminemia were managed as SRE. However, current guidelines for treating protein-losing enteropathy recommend using low-fat diets [58].

Previous studies have shown significant shifts in the intestinal microbiota of dogs with CIE. For example, reductions in beneficial bacteria such as *Akkermansia muciniphila* have been found both in the lumen and colonic crypts of affected dogs [19]. Meanwhile, an increase in luminal *Escherichia coli* [19] and a more significant number of Enterobacteriaceae with lower rates of Firmicutes and Bacteroidetes [34,35] are reported in the assessment of the intestinal microbiota of dogs with CIE. These findings suggest that microbial composition and function, rather than total bacterial load alone, may play a role in disease phenotype. Therefore, it is also possible that differences in specific bacterial taxa or microbial metabolites between response groups (FRE, ARE, and SRE) could explain variations in treatment outcomes. However, these factors were not assessed in the present study.

This study is limited by the lack of standardization of the diagnostic protocol due to its retrospective nature, involvement of multiple clinicians, and lack of standardized diagnostic and treatment protocols. The CIBDAI was retrospectively calculated for 22 out of 54 dogs, potentially affecting the accuracy of the assessment and study power. Additionally, duodenal and ileal samples, as well as long-term follow-up data, were not available for all dogs, further limiting the study’s power. While formalin is not ideal for preserving the mucus layer [41] and may have led to an underestimation of bacterial abundance [18], previous studies support the use of FISH on formalin-fixed biopsies from the small intestine of dogs [5], cats [59], and humans [60,61]. The use of formalin may affect attached bacteria, but its impact should be consistent across groups and would not influence invasive bacteria in the lamina propria. Further investigation into attached bacteria in formalin-fixed samples may enhance understanding of their clinical relevance in histopathologic diagnostics, since this is the fixative routinely used. Additionally, many of the dogs did not undergo more than one dietary trial. If they had responded to a second dietary trial, their CIE subtype classification would have changed to FRE [17]. Finally, the analysis was restricted to eubacteria, which may have limited the detection of disease-associated microbial signatures that could have been revealed by targeting specific bacterial taxa.

In summary, dogs with ARE, FRE, and SRE did not show increased bacterial loads in the duodenal mucosa with FISH. Although dogs with ARE showed a higher abundance of superficial and attached bacteria in the ileal mucosa compared to healthy controls, no significant differences were observed between the CIE categories. Moreover, summative histopathologic scores and select histopathologic parameters overlapped between the CIE groups. Taken together, these results suggest that histopathologic findings and fluorescence in situ hybridization results should not be used solely to predict treatment response in CIE but instead should be interpreted in light of clinical findings to guide treatment decisions.

## Supporting information

S1 Table Bacterial area in pixels corresponding to total bacteria and attached bacteria in duodenal and ileal biopsies from dogs with chronic inflammatory enteropathy (CIE) and healthy control dogs. (DOCX)

S1 Text Supporting information on fluorescence in situ hybridization and image analysis methods. (DOCX)
