# Supplementary material for: Abundance of bacteria and histopathologic findings in the small intestinal mucosa of dogs with chronic inflammatory enteropathies
Source: PLoS One. 2025 Dec 22;20(12):e0337930. doi: 10.1371/journal.pone.0337930 (PMC12721506; doi:10.1371/journal.pone.0337930)
Supplement: S1 Table — (DOCX) [file pone.0337930.s001.docx]

**Supporting Information**

**S1 Table**. **Bacterial area in pixels corresponding to total bacteria and attached bacteria in duodenal and ileal biopsies from dogs with chronic inflammatory enteropathy (CIE) and healthy control dogs.**

| Organ | Group | Total bacteria | Attached bacteria |
| --- | --- | --- | --- |
| Duodenum | Control | 93,5 (0-164,5) | 0 (0-83,5) |
|  | FRE | 36,5 (0-181,5) | 0 (0-32,5) |
|  | SRE | 34 (0-269,5) | 0 (0-38,5) |
|  | ARE | 32 (4-148,5) | 0 (0-37,5) |
|  |  |  |  |
| Ileum | Control | 3,75 (0-63) | 0 (0-0) |
|  | FRE | 123,5 (0-3249) | 0 (0-358) |
|  | SRE | 139 (0-393) | 44,75 (0-183,5) |
|  | ARE | 163 (0-1341,5)**^a^** | 108 (0-367,5)**^a^** |

Data expressed as median and range of bacterial area in pixels.

FRE, food-responsive enteropathy; SRE, steroid-responsive enteropathy; ARE, antibiotic-responsive enteropathy.

^a^ significant (*P* < 0.05) difference between CIE group and healthy control group
