# Supplementary material for: Abundance of bacteria and histopathologic findings in the small intestinal mucosa of dogs with chronic inflammatory enteropathies
Source: PLoS One. 2025 Dec 22;20(12):e0337930. doi: 10.1371/journal.pone.0337930 (PMC12721506; doi:10.1371/journal.pone.0337930)
Supplement: S1 Text — (DOCX) [file pone.0337930.s002.docx]

**S1Text. Supporting Information on protocols**

**Fluorescence in situ hybridization**

After deparaffinization in xylene and hydration in ethanol, sections were individually hybridized with an oligo probe diluted to 300 ng/mL in hybridization buffer (pH 7.2). The hybridization buffer consisted of Tris (0.242 g), NaCl (0.876 g), SDS (0.1 g), formamide (20 mL), dextran sulfate (10 g) in 80 mL of ultrapure water. The pH of the solution was adjusted to 7.2 with HCl.

The wash buffer consisted of Tris (2.42 g) and NaCl (8.76 g) in 1L of ultrapure water. The pH of the solution was adjusted to 7.2 with HCl.

The slides were maintained at 40˚C in a hybridization chamber consisting of a cover plate slide rack for sixteen hours and protected from light. After hybridization, slides were rinsed with a wash buffer and distilled water, followed by air drying. The slides were mounted with an antifade solution containing 4′,6-diamidino-2 phenylindole (DAPI), and coverslipped.

The hybridization temperature of 40°C, instead of the 46°C used in most protocols, was chosen because formamide was added to the hybridization buffer. Formamide lowers the melting point and annealing temperature of nucleic acid strands in situ hybridization. During optimization on positive control sections, slides hybridized at 40°C resulted in less background staining than those hybridized at 46°C, without compromising labeling of bacteria.

**Image analyses**

Under the fluorescence microscope, each field was captured with the DAPI filter for the identification of host cell nuclei, fluorescein isothiocyanate (FITC) filter for background autofluorescence from the host epithelium, and tetramethylrhodamine-isothiocyanate (TRITC) filter for identification of probe-labeled bacteria. Autofluorescence is frequently observed in fixed tissues. Erythrocytes and lipofuscin pigment exhibit high autofluorescence under FITC (green) and TRITC (red) filters. The images obtained with the three filters were merged for image analyses, so only pixels corresponding to labeled bacteria (orange to red) would be quantified. On merged images, autofluorescent erythrocytes and lipofuscin are yellow due to the merging of red and green signals.

Image analyses were performed using the software ImageJ as follow:

*Total bacteria:*

- Open the image to be analyzed (jpg format) > Select Image >Adjust > Color Threshold.


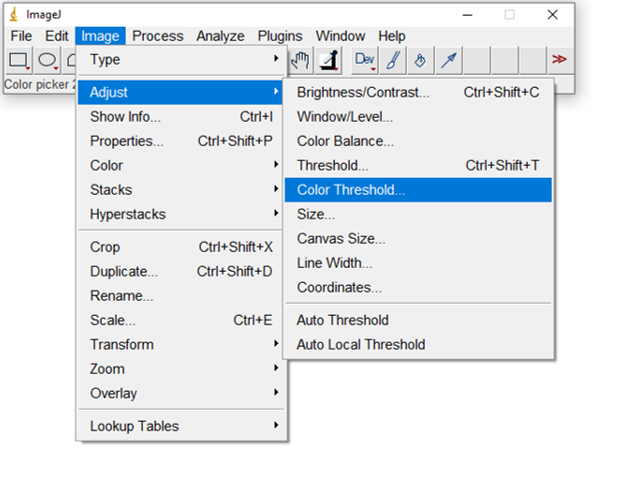


- A Threshold color menu will be opened. Set the thresholding method as Default and the threshold color as preferred (white is suggested). Select orange to red color hue corresponding to labeled bacteria.
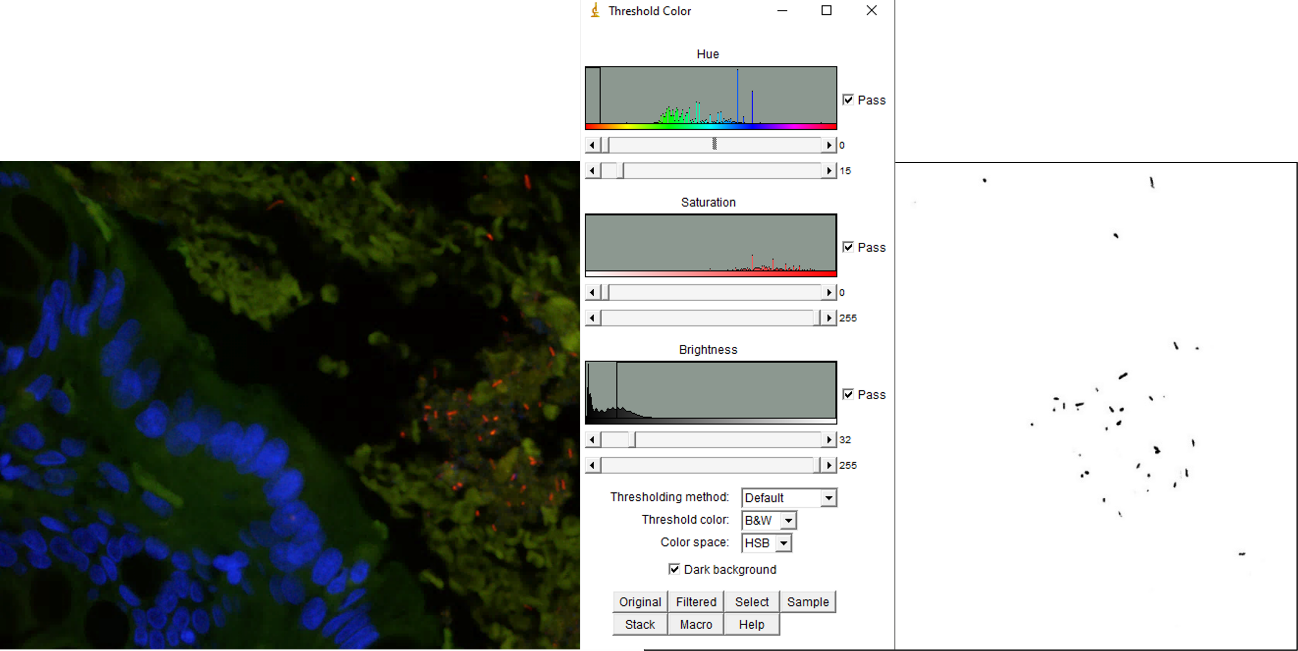

- Go to Analyze in the menu bar > Analyze particles > Select Summarize > Ok.

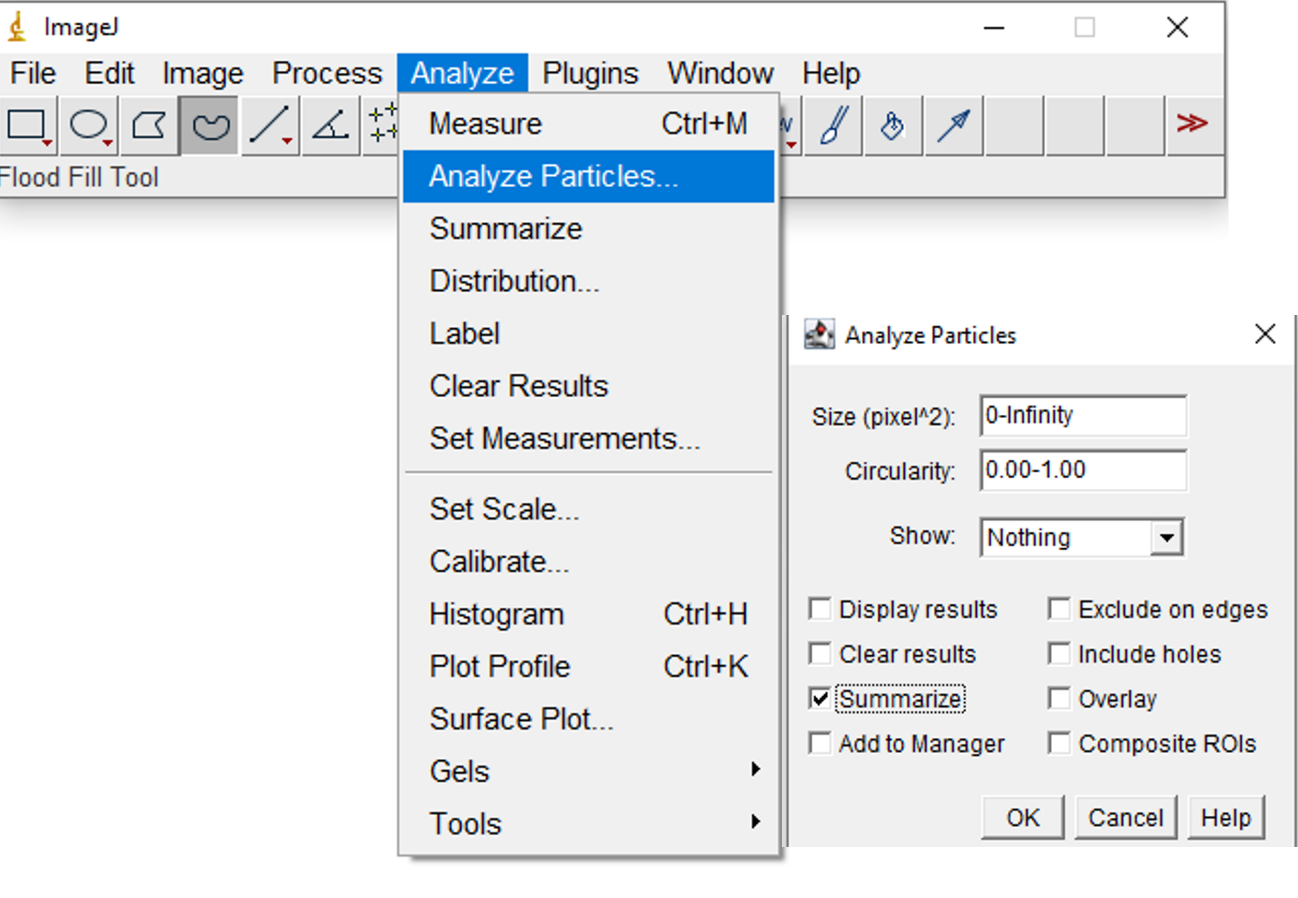

- A summary box will be opened and the data regarding the total area in pixels corresponding to bacteria (orange to red) will be shown.
- Repeat this process for all ten images of each slide/case and register the total area values to be used for calculation of the mean area of total bacteria for that slide/case.


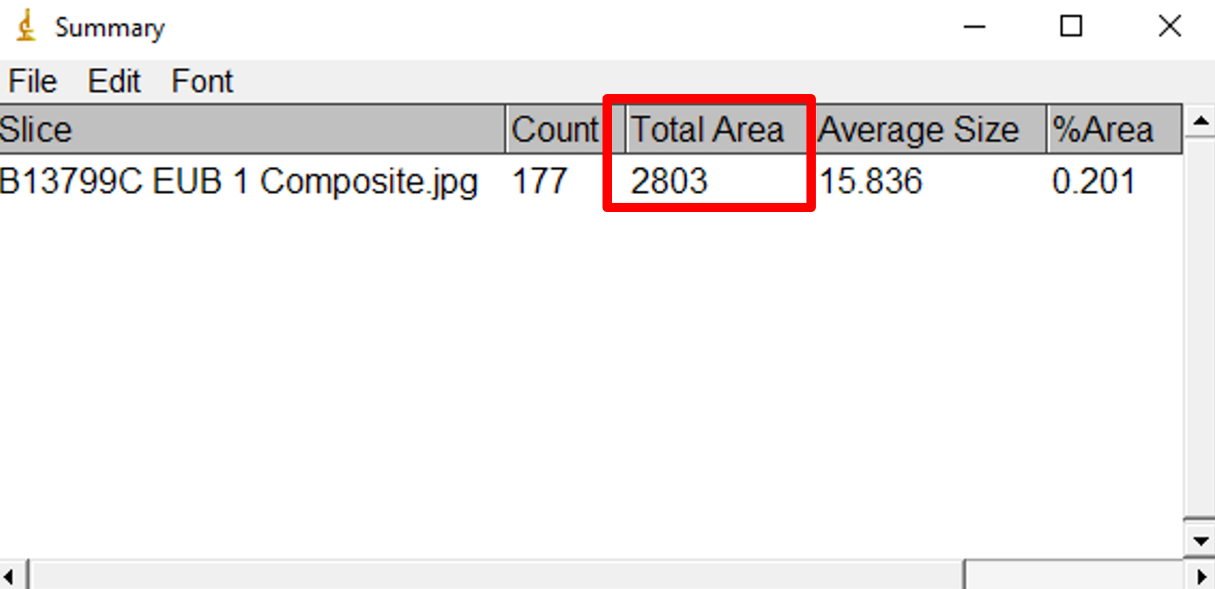


*Attached and invasive bacteria:*

- After registering the total labeled bacteria area for that image, manually select the areas with attached bacteria to be analyzed. For that, go to the freehand selections tool in the toolbar and select only the areas where bacteria are attached to the superficial epithelium.


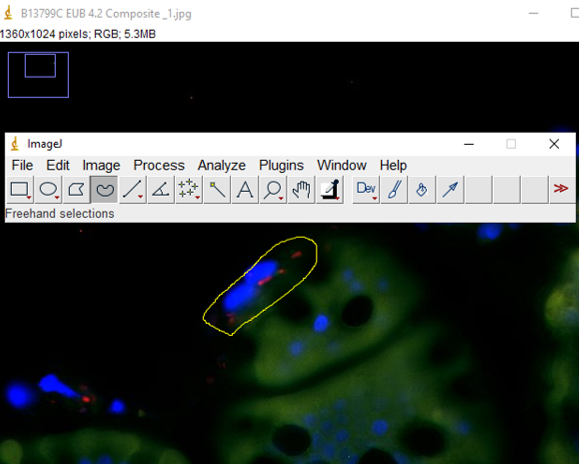


- Following the selection, repeat the “analyze particle” process described above. The total area displayed in the summary box will be the total area in pixels corresponding to the region of interest that has been manually selected.
- The value is then registered as the total area of attached bacteria
- Following the above instructions for attached bacteria, manually select the regions where there are invasive bacteria within the mucosa, if any, in that same image.
- Repeat the process for the ten images for the case/slide
- Calculate the mean area corresponding to attached bacteria for that case/slide.
